# Identification of Gene Modules Associated with Low Temperatures Response in Bambara Groundnut by Network-Based Analysis

**DOI:** 10.1371/journal.pone.0148771

**Published:** 2016-02-09

**Authors:** Venkata Suresh Bonthala, Katie Mayes, Joanna Moreton, Martin Blythe, Victoria Wright, Sean Tobias May, Festo Massawe, Sean Mayes, Jamie Twycross

**Affiliations:** 1 School of Computer Sciences, Jubilee Campus, University of Nottingham, Nottingham, United Kingdom; 2 School of Biosciences, University of Nottingham Malaysia Campus, Kuala Lumpur, Malaysia; 3 Crops For the Future, Jalan Broga, 43500 Semenyih, Malaysia; 4 Plant and Crop Sciences, Biosciences, Sutton Bonington Campus, Loughborough, Leicestershire, United Kingdom; 5 Advanced Data Analysis Centre, University of Nottingham, Sutton Bonington Campus, Loughborough, Leicestershire, United Kingdom; 6 School of Veterinary Medicine and Science, University of Nottingham, Sutton Bonington Campus, Loughborough, Leicestershire, United Kingdom; 7 Deep Seq, School of Life Sciences, University of Nottingham, Medical School, Queen’s Medical Centre, Nottingham, United Kingdom; New Mexico State University, UNITED STATES

## Abstract

Bambara groundnut (*Vigna subterranea (L*.*) Verdc*.) is an African legume and is a promising underutilized crop with good seed nutritional values. Low temperature stress in a number of African countries at night, such as Botswana, can effect the growth and development of bambara groundnut, leading to losses in potential crop yield. Therefore, in this study we developed a computational pipeline to identify and analyze the genes and gene modules associated with low temperature stress responses in bambara groundnut using the cross-species microarray technique (as bambara groundnut has no microarray chip) coupled with network-based analysis. Analyses of the bambara groundnut transcriptome using cross-species gene expression data resulted in the identification of 375 and 659 differentially expressed genes (p<0.01) under the sub-optimal (23°C) and very sub-optimal (18°C) temperatures, respectively, of which 110 genes are commonly shared between the two stress conditions. The construction of a Highest Reciprocal Rank-based gene co-expression network, followed by its partition using a Heuristic Cluster Chiseling Algorithm resulted in 6 and 7 gene modules in sub-optimal and very sub-optimal temperature stresses being identified, respectively. Modules of sub-optimal temperature stress are principally enriched with carbohydrate and lipid metabolic processes, while most of the modules of very sub-optimal temperature stress are significantly enriched with responses to stimuli and various metabolic processes. Several transcription factors (from MYB, NAC, WRKY, WHIRLY & GATA classes) that may regulate the downstream genes involved in response to stimulus in order for the plant to withstand very sub-optimal temperature stress were highlighted. The identified gene modules could be useful in breeding for low-temperature stress tolerant bambara groundnut varieties.

## Introduction

Bambara groundnut (*Vigna subterranea (L*.*) Verdc*, 2*n* = 2*x* = 22) is a nutritionally rich, underutilised, indigenous African legume crop and mainly grown for its protein rich seed. This crop continues to be the third most important food legume crop after groundnut and cowpea in semi-arid Africa [1, 2]. Due to its good seed nutritional values, particularly for the protein component in developing country diets, bambara groundnut has been receiving increased interest and could undergo a shift from subsistence crop to a cash crop i.e., bambara groundnut has been canned at a commercial level [3]. The remarkable feature of this crop is that it can produce yield in soils which are too poor for cultivation of other crops like groundnut (*Arachis hypogaea*) or where drought stress is too extreme [4].

Low temperature (LT) can be a major abiotic stress, particularly in non-equatorial regions of Africa and elevated planes. LT stress can limit the growth and development of the crop, leading to the loss of potential yield, with the limited reports for bambara groundnut suggesting base temperatures (below which no growth and development occurs) of 12–13°C. Similar to other crops [5,6], the growth and productivity of bambara groundnut is also effected by LT stress in a number of ways, such as delays in the germination of seeds, a reduction in total dry matter accumulated (TDM), reduced shelling percentage and reduced pod and seed yields. Usually the optimum temperature (T_0_) for bambara groundnut ranges from 20–28°C [4, 7]. Thus, the low temperatures restrict the times of the year when the farmers can plant seed, particularly where the temperature at night is below the T_0_. However, while landraces which are grown within such stressed environments have been indirectly selected by growth in the target environment, any cold tolerance present may not be optimised and without an understanding of the trait, it may be difficult to introduce new genetic variation from other environments into the target environment. Despite its importance as a promising food and economically valuable crop, bambara groundnut is mainly cultivated as landraces and there is a need to develop bambara groundnut varieties which are tolerant to low temperature stress, which could further improve the options for growth and productivity of this drought tolerant legume as part of sustainable low input agriculture.

The advancement of molecular technologies and high-throughput “omics” tools such as microarrays and deep-sequencing studies have become a useful strategy for the global analysis of plant gene expression under a particular biotic and/or abiotic stress condition. Using microarrays, the abiotic stress responses in *Arabidopsis thaliana* [8], *Oryza sativa* [9], *Glycine max* [10], *Solanum lycopersicum* [11] and in other plants have been widely analyzed and several stress responsive genes have been identified, although these genes have yet to be evaluated in field crop situations. Unfortunately, commercial microarray chips are not available for underutilized and understudied minor crops like bambara groundnut and alternatives such as comprehensive RNA sequencing—while becoming more affordable—can still be costly, prohibiting routine large-scale experiments. One possible solution for this problem is a cross-species (Xspecies) microarray approach [12], involving the hybridisation between the cRNA of the species of interest and a closely related species for which a custom microarray chip is available. This approach has been successfully used to study the transcriptomes of different important crops [13–15]. However, these microarray analyses may provide less information on gene-gene functional relationships. To study the gene-gene interrelationships under a particular biological treatment, such as experiencing biotic and/or abiotic stress conditions, network-based analyses, such as gene co-expression network analyses, have been popularly used [16–20].

Gene co-expression networks are constructed from expression data generated by using either microarrays or deep-sequencing (RNA-Seq) methods to provide a global view of gene-gene interrelationships and can help in the identification of candidate genes as well as the better understanding of how the inter-connected genes interplay to carry out specific biological functions under a specific stress condition. In recent years, co-expression networks have been constructed for model species, *Arabidopsis* [16], and for other important food crops such as *Oryza sativa* [17], *Citrus* [18], *Glycine max* [19], *Vitis vinifera* [20] and have been provided a global view of the transcriptional relationships under specific stress conditions. From these gene co-expression network analyses, a number of candidate genes and gene functional modules have been identified that are associated with a specific biological process in plants. In addition, several web-based databases for plant gene co-expression networks, including LegumeGRN [21], CoP [22], PLANEX [23], PLEXdb [24] and CressExpress [25], have been developed to enable the visualization of gene-gene interrelationships, gene-specific expression profiles across stress conditions and data mining of co-expression networks for plant breeders and biologists.

In the present study we report the use of cRNA from bambara groundnut grown under sub-optimal and very sub-optimal temperature stresses, hybridized with Affymetrix Soybean's GeneChip^TM^ array to study low temperature responsive candidate genes expression in bambara groundnut. To further study gene-gene functional relationships and identify the gene functional modules associated with LT, we have constructed a gene co-expression network, followed by the partitioning of the co-expression networks to identify LT responsive functional modules to provide a better understanding of the underlying molecular mechanisms of the LT response in bambara groundnut.

## Materials & Methods

### Plant Materials

Three plants of the bambara groundnut genotype ‘S19-3’ were grown in controlled environment growth rooms at Sutton Bonington Campus, University of Nottingham, under a 12 hour photoperiod and at a constant temperature of 27°C. Plants were grown in soil columns containing a growing medium of 1 part John Innes 2 compost to 1 part sand, and were watered as required to main approx. field capacity. A single, fully-expanded leaflet was sampled from each of the three plants growing at 27°C, snap frozen in liquid nitrogen and stored at -80°C. Plants were given a further 3 days at 27°C to recover from sampling before being moved to a controlled environment room at 23°C. On the fifth day at 23°C a single fully-expanded leaflet was sampled and stored, as described above. After 3 further days plants were moved to 18°C, and then five days later were sampled again.

### Genomic DNA and total RNA extraction

Frozen bambara groundnut leaflets were ground to a fine powder, under liquid nitrogen, in a mortar and pestle. Extraction of genomic DNA was carried using 100 mg ground leaf tissue, using a DNeasy Plant Mini Kit (Qiagen), according to manufacturer’s directions. The quality and concentration of DNA was assessed by visualization on agarose gel and by spectrophotometry, using a nanodrop. For extraction of total RNA, 100 mg ground leaf tissue was processed, as per manufacturer’s instructions, using an RNeasy Plant Mini Kit (Qiagen) and including the additional DNA digestion step. RNA samples were then run on the Agilent Bioanalyzer to determine quality and concentration.

### Genomic DNA and cRNA hybridization

Genomic DNA from bambara groundnut leaf tissue was labelled with the Bioprime DNA labelling system kit and then hybridized with Soybean’s GeneChip^TM^ (Affymetrix) for 16 hours at 45°C using the standard hybridization protocol (Affymetrix) and analysed by scanning. Total RNA (5 μg) from bambara groundnut of optimal, sub-optimal and very sub-optimal temperatures was reverse-transcribed to synthesize double stranded cDNA using the standard protocol and then the resulting samples were *in-vitro* transcribed to generate complementary RNAs (cRNAs) incorporating biotinylated nucleotides using T7 DNA polymerase. Purified cRNA (15 μg) were heat-treated and then hybridized to the Soybean GeneChip^TM^ (Affymetrix) for 16 hours at 45°C [12]. A triplicate hybridization (biological, from different plants) was used for each temperature (18°, 23° and 27°C) and all the hybridization data have been submitted to NCBI’s GEO database (https://www.ncbi.nlm.nih.gov/geo; Acc. No. GSE72255).

### Probe selection and Normalization

Probe-pair information from the genomic DNA (gDNA) hybridization file (.cel) were extracted using a.cel file parser available at https://www.affymetrix.arabidopsis.info/xspecies/, which generates a customised chip definition file (CDF) compatible with various microarray data analysis programs, including R/BioConductor[12]. The RNA hybridisation files (.cel) were loaded into the R environment applying the customized CDF files generated in the previous step and then were pre-processed using the RMA (Robust Multichip Average) pre-normalization algorithm present in the “affy” package [26].

### Identification of differentially expressed genes

Differentially expressed genes were identified based on the baseline data generated under 27°C (control) in both sub-optimal (23°C) and very sub-optimal temperature (18°C) stresses using a t-test and corrected by False Discovery Rate (FDR) approach for multiple hypothesis testing [27]. Average signal intensities of all biological replicates for each sample were used to calculate the fold-change of gene expression.

### RNA-seq data based expression profiling of DE genes

Unpublished SOLiD4 reads were generated from the same RNA samples as the microarray data and have been submitted to NCBI’s GEO database (https://www.ncbi.nlm.nih.gov/geo; Acc. No. GSE75982). Reads which were at least 50bp in length, were aligned to an unpublished transcriptome assembly of Bambara groundnut using LifeScope version 2.5.1 [28] and read counts were extracted from the alignment file. DESeq package version 1.22.0 [29] were used to identify the differentially expressed genes. Further, the expression patterns of DE genes identified from RNA-seq data were matched with the DE genes identified from microarray data via BLASTN [30] searching of bambara groundnut’s unpublished transcriptome sequences against the Soybean GeneChip design sequences with default parameters. The matched differentially expressed probes were visualised, based on variance stabilization transformed data, using heatmaps by heatmap.2 function of R’s gplots package version 2.1 (http://cran.r-projects.org/package=gplots).

### Construction of the co-expression network

Highest-reciprocal rank (HRR) based co-expression network methodology was employed to further investigate the interactions between the identified DE genes by constructing co-expression networks for each of the stress conditions. The HRR-based co-expression network were created using the HRRNetworkCreator tool [31] and then the co-expression networks were visualized using Cytoscope [32].

### Module detection and Functional enrichment

HCCA [31] was used to detect the modules in the constructed co-expression networks using default parameters. The GO annotation of the *Glycine max* genome was downloaded from SoyBase (http://www.soybase.org). Significantly enriched Gene Ontology terms for each of the detected module was carried out by AgriGO Tool [33].

## Results

**Fig 1:**
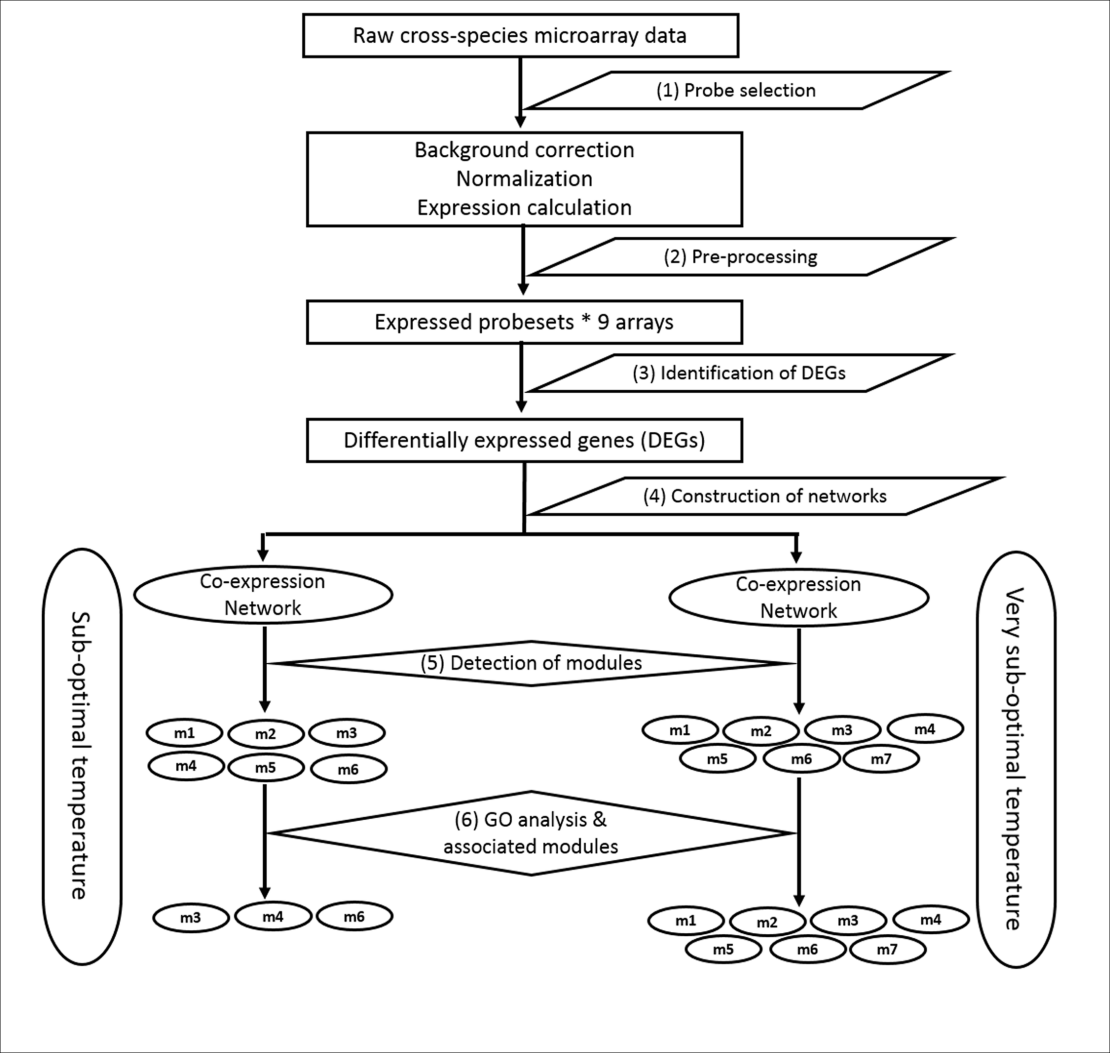
Flowchart of the pipeline for identification of gene modules associated with low temperature response in bambara groundnut.

Both soybean and bambara groundnut are phylogenetically related legume crops (around 20 million years divergence since a common ancestor; [34]). Hence in this study we have used Soybean's GeneChip^TM^ array (Affymetrix) to study the transcriptome of Bambara groundnut under low temperatures. Further, we have developed a computational pipeline (Fig 1) to identify the low temperature stress associated genes/gene modules using cross-species based expression datasets coupled with network-based analysis. The pipeline consists of the following steps: (1) probe selection using a gDNA hybridization approach, (2) pre-processing of raw expression data, (3) identification of DE genes, (4) construction of an HRR-based co-expression network, (5) detection of modules, and (6) detection of biologically relevant modules by GO analysis.

### Probe Selection and identification of differentially expressed genes

After hybridizing the genomic DNA of bambara groundnut with the Soybean GeneChip^TM^ array (Affymetrix), a probe-pair was retained if its perfect-match hybridization intensity value was greater than the user defined threshold values (ranging from 0 to 500) using a.cel file parser, which also generated a CDF file for each of the threshold values. These CDF files only contain the probe-pairs in which the perfect-match (PM) oligonucleotides has a gDNA hybridization intensity greater than the user defined threshold [12]. Table 1 shows the number of retained probe pairs and probe-sets for each threshold value for further analysis.

**Table 1:** Statistics of retained probes and probe-sets on the Affymetrix Soybean GeneChip array before further analysis. This table contains information on the number of probe-pairs and probe-sets retained for further analysis corresponding to each threshold value. The last two columns indicate the number of identified DEGs under sub-optimal and very sub-optimal temperature stresses compared to the optimal temperature, corresponding to each threshold value, based on masked probe-sets in the custom.cdf file.

Threshold values	Number of probes (complete Soybean chip)	Number of probesets (complete Soybean chip)	Number of DEGs (Sub-optimal temperature)	Number of DEGs (Very sub-optimal temperature)
0	670388	61072	256	513
50	602176	61058	342	612
100	326207	59978	375	659
200	130572	50822	335	592
300	65127	36716	254	372
400	36933	25017	190	238
500	23184	17163	127	192

The raw expression intensity values (RNA.cel files) were filtered for low- or non-specific hybridization using the custom generated CDF files from the previous step and then normalized using the RMA algorithm present in “affy” package of BioConductor in an R environment [26]. To further reduce the noise in the normalized data, only the probe-sets present (P) in all the array slides (total 9 slides) were selected. Differentially expressed genes (DEGs) were calculated for each threshold value (0–500) using a t-test and then corrected by using False Discovery Rates (FDR) for multiple hypothesis testing [27]. To consider a probe-set as differentially expressed, it should contain <0.01 corrected p-value with a >2 fold-change (either up- or down-regulated). Based on this criteria, the CDF file of 100 threshold values was considered as a best threshold value (Table 1) as it returned highest number of DEGs (375 in optimal vs. sub-optimal temperature and 659 in optimal vs. very sub-optimal temperature). Out of 375 DEGs, 204 genes are up-regulated while 171 genes are down-regulated under the sub-optimal temperature (S1 Table). On the other hand, out of 659 DEGs, 403 genes are up-regulated while 257 genes are down-regulated in the very sub-optimal temperature (S2 Table). There were 110 DEGs which are common between sub-optimal and very sub-optimal temperatures suggesting that a similar genetic response is likely to underlie the response of bambara groundnut to these two different temperatures. Out of these 110 common DEGs, 76 genes are up-regulated while 34 genes are down-regulated (S3 Table).

### RNA-seq data based expression profiling of DE genes

We have chosen top hundred DE genes (up- and down-regulated) to validate their expression patterns using unpublished RNA-seq data in three steps: (1) Aligned RNA-seq reads of three stress conditions individually to the unpublished transcriptome assembly of bambara groundnut using LifeScope version 2.5.1 [28] and read count for each of the isotig were extracted; (2) Then the count data were load into DESeq [29] and identified DE genes with adjusted p-value < 0.1; and (3) The top hundred DE genes (up- and down-regulated) of microarray data were matched against the DE genes obtained from RNA-seq data via BLASTN [30] searching of isotigs of bambara groundnut’s transcriptome against the Soybean GeneChip design sequences with default parameters which resulted in 52 common DE genes (up- and down-regulated) between microarray-based top hundred DE genes and RNA-seq data based DE genes (adjusted p-value <0.1). Finally, the 52 common DE genes were used to generate the heatmap (S1 Fig and S4 Table) using heatmap.2 function. The heatmap indicates that the 52 DE genes identified from microarray data were consistent when using either cross-species microarray approaches or RNA-seq based analysis.

### Construction of co-expression networks and detection of functional modules

We have employed the Highest Reciprocal Rank (HRR) based method to construct the co-expression networks. In this approach, the calculated Pearson Correlation Coefficient (r) values were normalized using the highest reciprocal rank i.e., the mutual co-expression relationship between two genes of interest. This method overcomes the problem of calling biological relationships between expressed genes at different cut-off r-values [31]. In general, the statistical significance of the co-expression relationships between two genes of interest may not reflect the biological relevance [35]. Therefore, we determined HRR value that optimized biological relevance and found that the 10 ≤ HRR ≤ 20 produced biologically relevant networks in both the stress conditions i.e., HRR = 10 produced 28% and 34% of the nodes disconnected from networks of sub-optimal and very sub-optimal temperature stress conditions, respectively, while HRR = 20 decreased this proportion to 0.8% and 0.3% of nodes, respectively. Thus, combining both biological relevance and selecting for the maximum number of nodes connected in both the networks, we found that HRR = 20 resulted in biologically relevant co-expression networks with 4218 edges between 375 nodes (S5 Table) and 7178 edges between 659 nodes (S6 Table) in sub- optimal and very sub-optimal temperatures, respectively.

Responses to any stress are usually organized as relatively separable functional modules of highly interconnected genes in the co-expression network at a given cut-off. Genes present in the same functional module are co-expressed across diverse conditions, and thus, functional consistency among the genes present in the same modules is expected [17, 36, 37]. In this study we used the Heuristic Cluster Chiseling Algorithm (HCCA) [31], with the number of steps away from the seed node (n) = 3 and the average cluster size ranging from 40–200 nodes, to detect biologically related modules and this resulted in 6 (S7 Table) and 7 (S8 Table) modules in sub-optimal and very sub-optimal temperatures, respectively, ranging in size from 40–200 genes per cluster. Tables 2 & 3 shows the statistics of the detected modules in sub-optimal and very sub-optimal temperature co-expression networks, respectively.

**Table 2:** Summary of the detected modules in the sub-optimal temperature co-expression network. Module column indicates the module number in the network; The Total Genes column indicates the number of genes present in the module, with direction of gene expression change given in the next two columns.

Module	Total Genes	Up-regulated	Down-regulated
1	58	28	30
2	44	21	23
3	58	29	29
4	72	41	31
5	90	54	36
6	53	30	23

**Table 3:** Summary of the detected modules in the very sub-optimal temperature co-expression network. Module column indicates the module number in the network; The Total Genes column indicates the number of genes present in the module, with direction of gene expression change given in the next two columns.

Module	Total Genes	Up-regulated	Down-regulated
1	155	93	62
2	185	103	82
3	87	53	34
4	59	37	22
5	86	56	30
6	42	30	12
7	42	27	15

### Functional enrichment of detected modules

Gene Ontology (GO) annotation is one important steps in network analysis to understand the biological functions and then the identification of over-represented biological processes can reveal the functional features of each detected module. To GO annotate, followed by identifying the over-represented GO terms in the detected modules, we carried out a GO enrichment analysis using the AgriGO tool with the Soybean genome locus annotation of Phytozome as the reference set [33]. Hypergeometric distribution adjusted by Bonferroni correction for the testing of multiple hypotheses with an adjusted threshold of p<0.01 and a minimum number of mapping entries of 10 were used to evaluate the statistical significance of the functional enrichment within the detected gene modules.

#### Modules associated with sub-optimal temperature

**Fig 2:**
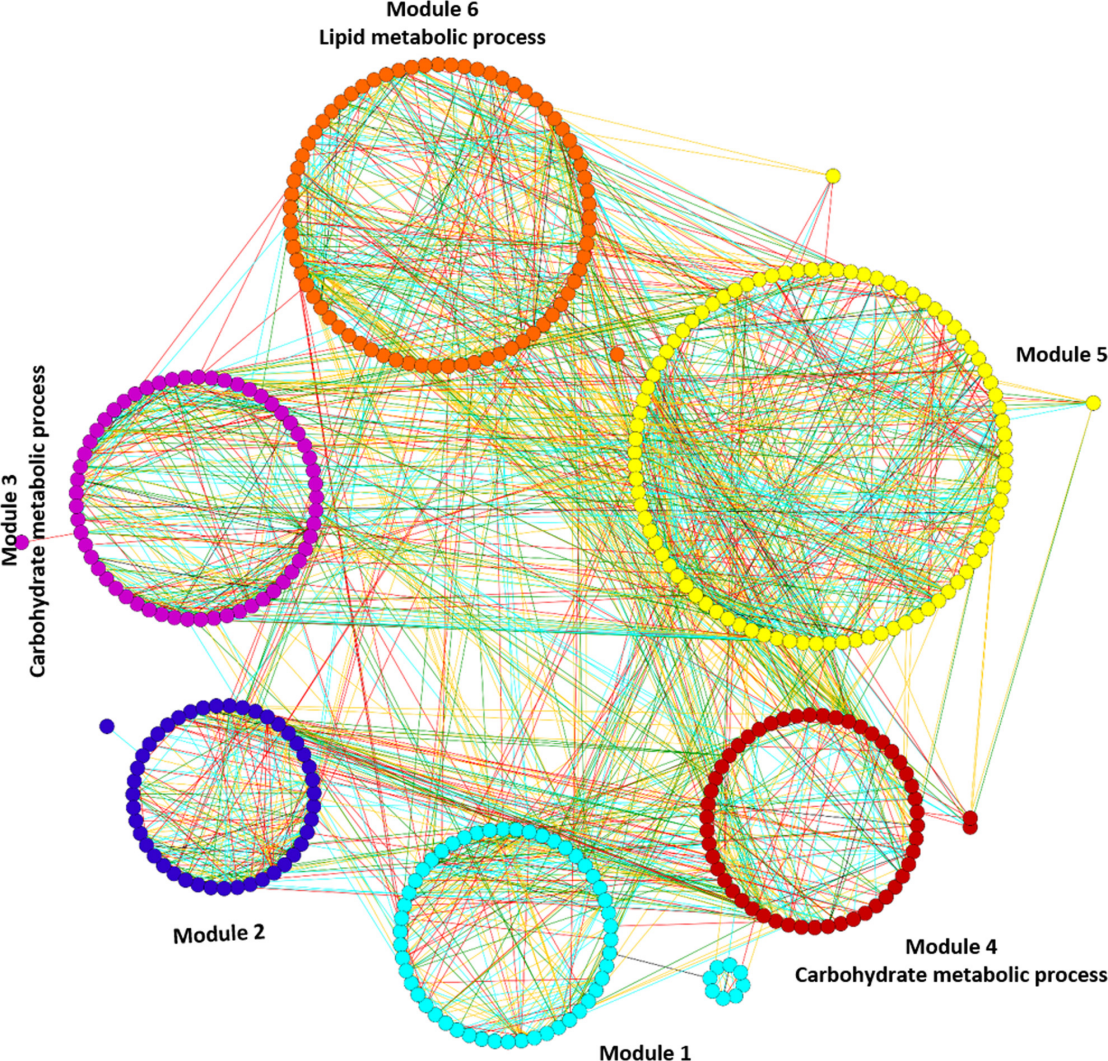
Mapping of enriched GO terms on the sub-optimal temperature stress responsive gene co-expression network. Modules 3, 4 and 6 were significantly enriched with GO terms. The different edge colors indicates different range of HRR values and as follows: Cyan: HRR1-HRR5; Red: HRR6-HRR10; Green: HRR11-HRR15 and Orange yellow: HRR16-HRR20.

Functional enrichment analysis resulted in the significant enrichment of three modules (3rd, 4th & 5th) with GO terms with the genes present in these modules principally involved in the biological processes of carbohydrate and lipid metabolic processes (S9 and S10 Tables). Fig 2 displays mapping of significantly enriched GO terms on the sub-optimal temperature stress responsive gene co-expression network.

Module 3 consists of 58 gene nodes and is principally enriched with carbohydrate metabolic processes (GO:0005975, FDR = 0.0023). There are a total of 12 genes whose functions are associated with carbohydrate metabolic processes, which represent nearly 28% of the annotated genes in the module. Interestingly, 50% of genes in this module are up-regulated by sub-optimal temperature stress, while another 50% of genes are down-regulated. Module 4 consists of 72 gene nodes and is also principally enriched with carbohydrate metabolic processes (GO:0005975, FDR = 0.00015). There are a total of 15 genes whose functions are associated with carbohydrate metabolic processes, which represents nearly 24% of the annotated genes in the module. Fifty-seven percent of genes are up-regulated in this module and the rest of the genes are down-regulated by sub-optimal temperature stress. In contrast, module 6 consists of 53 gene nodes and is principally enriched with lipid metabolic process genes (GO:0006629, FDR = 0.004). There are a total of 10 genes whose functions are associated with lipid metabolic processes, which represents nearly 24% of the annotated genes in the module. Fifty-seven percent of genes are up-regulated and the rest of the genes are down-regulated by sub-optimal temperature stress. Overall, 50–57% of genes in all the enriched modules were up-regulated, while 43–50% of genes are down-regulated by sub-optimal temperature stress, which indicates that the sub-optimal temperature stress has little effect on alteration in expression of genes associated with various metabolic enzymes of carbohydrate and lipid metabolic processes only.

#### Modules associated with very sub-optimal temperature

**Fig 3:**
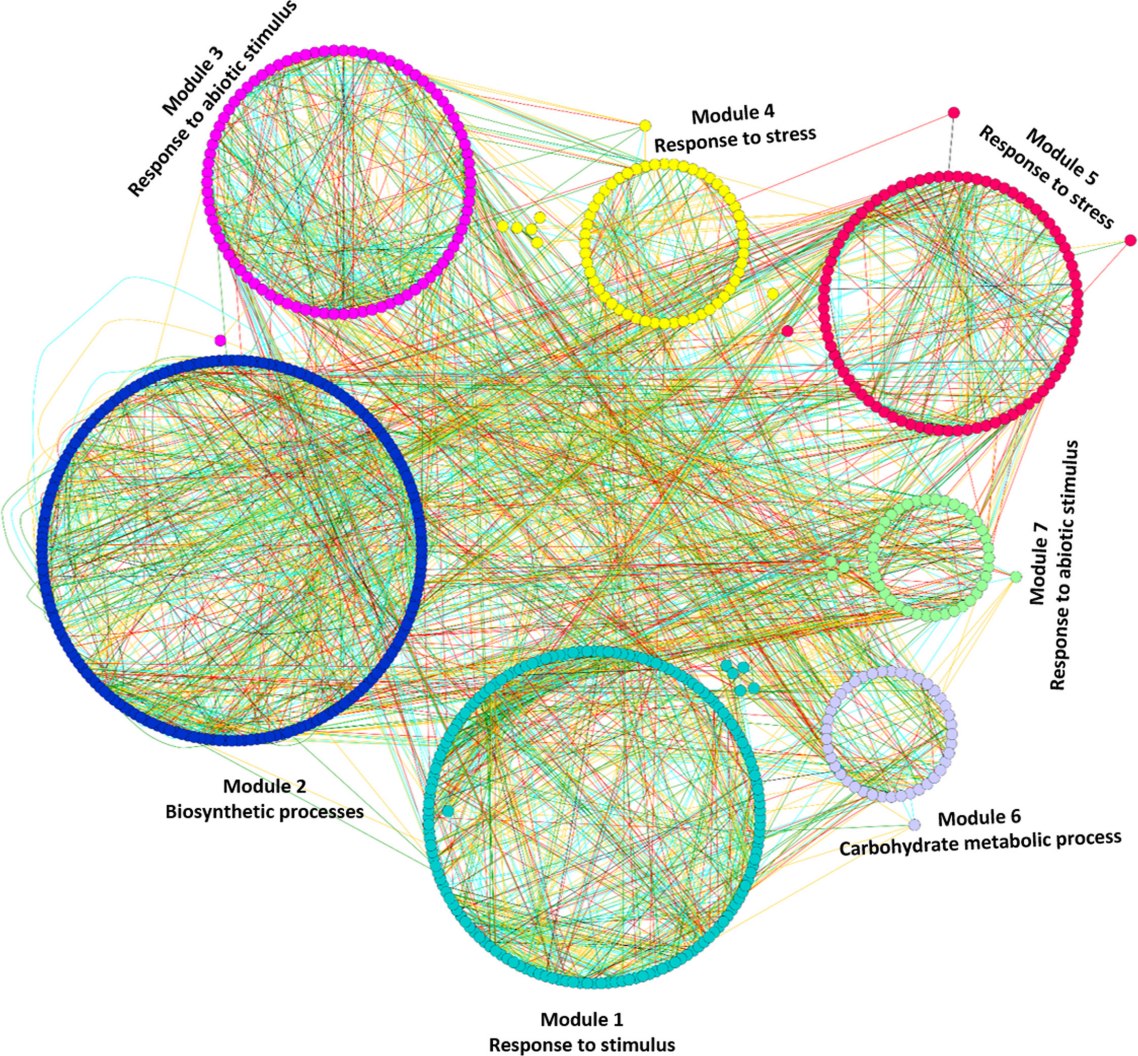
Mapping of enriched GO terms onto the very sub-optimal temperature stress responsive gene co-expression network. Modules with different colours indicates that these modules were significantly enriched with GO terms. The different edge colors indicates different range of HRR values and as follows: Cyan: HRR1-HRR5; Red: HRR6-HRR10; Green: HRR11-HRR15 and Orange yellow: HRR16-HRR20.

Functional enrichment analysis revealed that the very sub-optimal temperature stress responsive module genes were mainly involved in biological processes of various kinds of stress response, photosynthesis and cell and metabolic processes, including carbohydrate metabolism. In the very sub-optimal temperature all the detected modules were significantly enriched for GO terms (S11 and S12 Tables). Fig 3 displays mapping of enriched GO terms in the very sub-optimal temperature stress responsive gene co-expression network. Unlike the sub-optimal temperature stress, the very sub-optimal temperature stress regulated different kinds of stimulus responses along with cell and metabolic processes, which indicates that the plant responds to the decrease in temperature by altering more than one biological process in order to survive under unfavourable temperatures. All the detected modules in very sub-optimal temperature stress are positive regulators for low temperature stress in bambara groundnut.

Module 1 consists of 155 gene nodes and is enriched with stimulus responses (GO:0050896, FDR = 0.00039). There are a total of 67 genes whose functions are associated with responses to stimuli, which represent 54% of the annotated genes in the module. 60% of genes in the module are up-regulated by the very sub-optimal temperature stress, while rest of the genes are down-regulated. The presence of the up-regulated heat-shock protein (HSP) gene (Gma.10282.2.S1_at, Glyma18g01416.1) is indicative among the 67 genes enriched which show upregulation with the “stimulus response” in this module. In general, HSP genes are induced and secrete HSPs when there is sudden change in the genotypic expression of various genes triggered by any kind of stress. HSPs play an essential role as chaperones by assisting the correct folding of stress-accumulated misfolded proteins enhancing cell and plant survival during low temperature stress. It has also been reported that these HSP genes are induced in *Arabidopsis* and other plant species by low temperature stress [38] and thus this module may be preferentially associated with very sub-optimal temperature stress response in bambara groundnut.

Module 3 consists of 87 gene nodes and is enriched with abiotic stimulus response genes (GO:0009628, FDR = 0.0042). There are a total of 22 genes whose functions are associated with responses to abiotic stimuli, which represents 20% of the annotated genes in the module. Nearly 61% of genes in the module are up-regulated by the very sub-optimal temperature, while the rest of the genes are down-regulated. The up-regulated chaperone DnaJ gene is important among these 22 genes. Recent studies indicates that this gene contributes to maintenance of photosystem-II under chilling stress in tomatoes [39] and therefore, this gene may be participating in a similar response in bambara groundnut under very sub-optimal temperature.

Module 4 consists of 59 gene nodes and is enriched with responses to stress (GO:0006950, FDR = 0.0032). There are a total of 22 genes whose functions are associated with stress responses, which represents 47% of the annotated genes in the module. 63% of genes in the module are up-regulated, while the rest of the genes are down-regulated by the very sub-optimal temperature stress. This module contains an important up-regulated gene called WD40 repeat gene (Gma.13316.2.S1_a_at, Glyma18g52040.1) which a plays crucial role in diverse protein-protein interactions by acting as a scaffolding molecules and thus assisting the proper molecular activity of proteins. In recent studies, these genes show higher expression during a long duration of cold stress in *Setaria italica*, indicating the up-regulation of these genes in bambara groundnut may play a role in correct scaffolding of low temperature stress-accumulated misfolded proteins [40].

Module 5 consists of 86 gene nodes and is also enriched with responses to stress (GO:0006950, FDR = 0.0019). There are a total of 29 genes whose functions are associated with stress responses, which represents 46% of the annotated genes in the module. 65% of genes in the module are up-regulated, while the rest of the genes are down-regulated by the very sub-optimal temperature stress.

Module 7 consists of 42 gene nodes and is also enriched with abiotic stimulus response genes (GO:0009628, FDR = 0.0044). There are a total of 13 genes whose functions are associated with the abiotic stimulus responses, which represents 37% of the annotated genes in the module. Sixty-five percent% of genes in the module are up-regulated, while rest of the genes are down-regulated by the very sub-optimal temperature stress.

#### Transcription factors associated with the very sub-optimal temperature stress

Transcription factors (TFs) play a key role in the biological processes involved in plant growth and development, biosynthetic pathways and biotic and abiotic stress tolerances by regulating the downstream target genes. Recent studies have revealed their role in various biological processes in *Glycine max* [41, 42], *Brassica rapa* [43, 44], *Brassica oleracia* [45], *Vitis vinifera* [46] and *Setaria italica* [47–51]. In this study, we have identified several TFs only in the very sub-optimal temperature stress which may be regulate downstream target genes involved in stimulus responses contained in the GO term and the details of identified TFs are displayed in Table 4.

**Table 4:** Details of identified Transcription Factors (TFs). This table contains information for each identified TF with respect to the module number to which the TF belongs, the corresponding probe-set and gene ID, description of the TF, whether the identified TF is up- or down-regulated, the enriched GO term identified by the AgriGO tool and the number of genes connected to each TF.

Module	Probeset ID	Gene ID	Description	Regulation	Enriched GO Term	Intramodular connectivity
1	Gma.4281.1.S1_at	Glyma18g44560.1	WRKY70	Down	response to stimulus (GO:0050896)	11
1	Gma.593.2.S1_a_at	Glyma17g10250.2	MYB173	Down	response to stimulus (GO:0050896)	15
1	GmaAffx.36677.1.S1_at	Glyma04g33210.1	MYB16	Down	response to stimulus (GO:0050896)	14
1	Gma.4324.3.S1_a_at	Glyma20g31210.4	NAC17	Up	response to stimulus (GO:0050896)	5
2	GmaAffx.32030.1.A1_at	Glyma04g08990.1	GATA9	Down	response to stimulus (GO:0050896)	14
2	GmaAffx.20956.1.S1_at	Glyma10g05560.1	MYB118	Up	response to stimulus (GO:0050896)	13
3	Gma.13676.1.A1_at	Glyma03g41270.1	WHIRLY2	Up	response to stimulus (GO:0050896)	18
3	GmaAffx.50811.2.S1_at	Glyma15g08480.4	NAC73	Up	response to stimulus (GO:0050896)	18
7	GmaAffx.16512.1.S1_at	Glyma13g09010.1	MYB25	Up	response to stimulus (GO:0050896)	9

**Fig 4:**
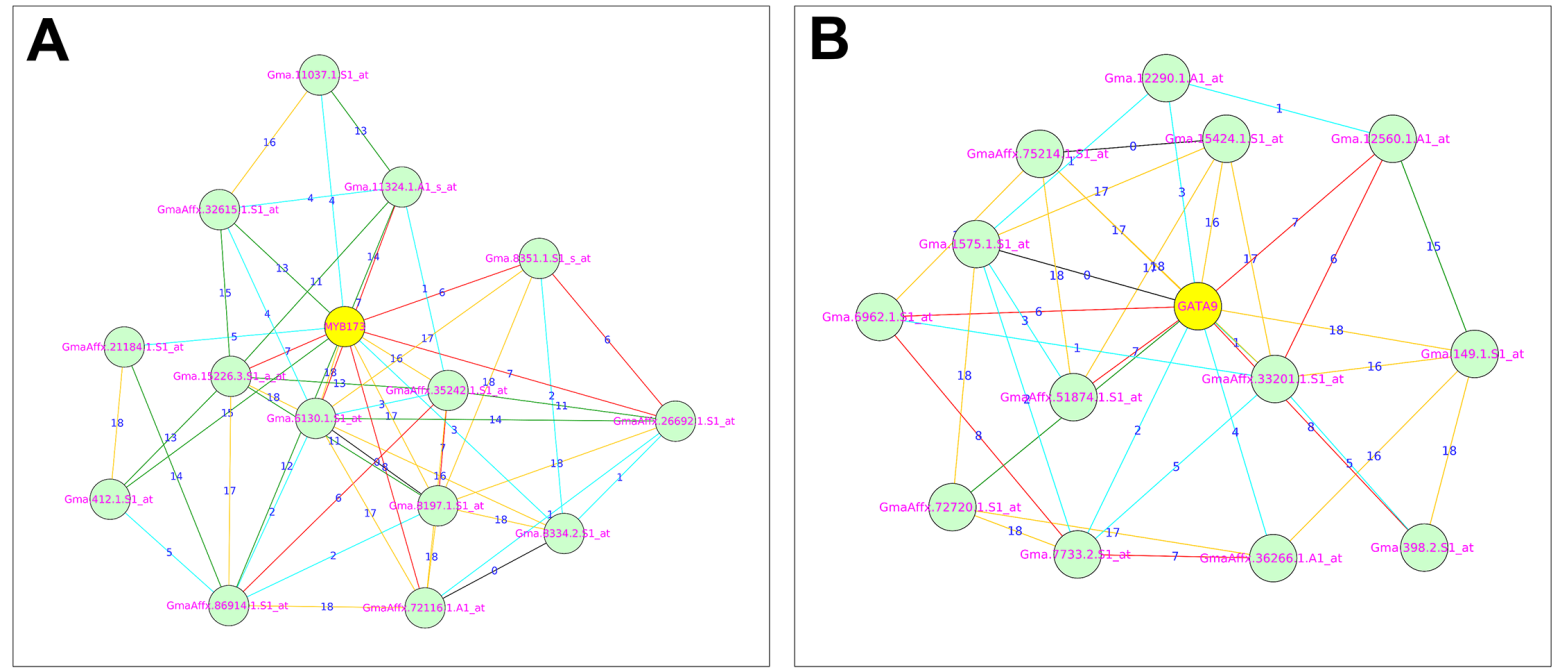
Co-expression relationships of MYB173 and GATA9 transcription factors with their related genes: [A] Shows the co-expression relationships of MYB173 with its related genes. [B] Shows the co-expression relationships of GATA9 with its related genes. The numbers on top of edges indicates the highest reciprocal rank between node genes. The different edge colors indicates different range of HRR values and as follows: Cyan: HRR1-HRR5; Red: HRR6-HRR10; Green: HRR11-HRR15 and Orange yellow: HRR16-HRR20.

Module 1 contains four genes encoding TFs from three families and are MYB16 (Glyma04g33210.1), MYB173 (Glyma17g10250.2), NAC17 (Glyma20g31210.4) and WRKY70 (Glyma18g44560.1). Out of four TFs, three TFs were down-regulated, while one TF is up-regulated by the very sub-optimal temperature stress. MYB173 is down-regulated by the very sub-optimal temperature stress and had the highest number of node genes connected (S13 Table) in module 1 (Fig 4A). Previous studies revealed that the MYB family TFs were induced in various stress responses and in stress tolerance [43, 49, 52] which indicates that the down-regulated MYB family TFs in bambara groundnut needs to be induced in order to withstand the decreasing temperature. Module 2 contains two genes encoding TFs from two families and are GATA9 (Glyma04g08990.1) and MYB118 (Glyma10g05560.1). GATA9 is down-regulated, while MYB118 is up-regulated by the very sub-optimal temperature stress. GATA9 had the highest number of intramodular connections (S13 Table) in module 2 (Fig 4B). Previous studies have revealed that the GATA family TFs were differentially expressed in high temperatures and drought [53] which indicates that GATA9 is induced in response to the decreasing temperature.

**Fig 5:**
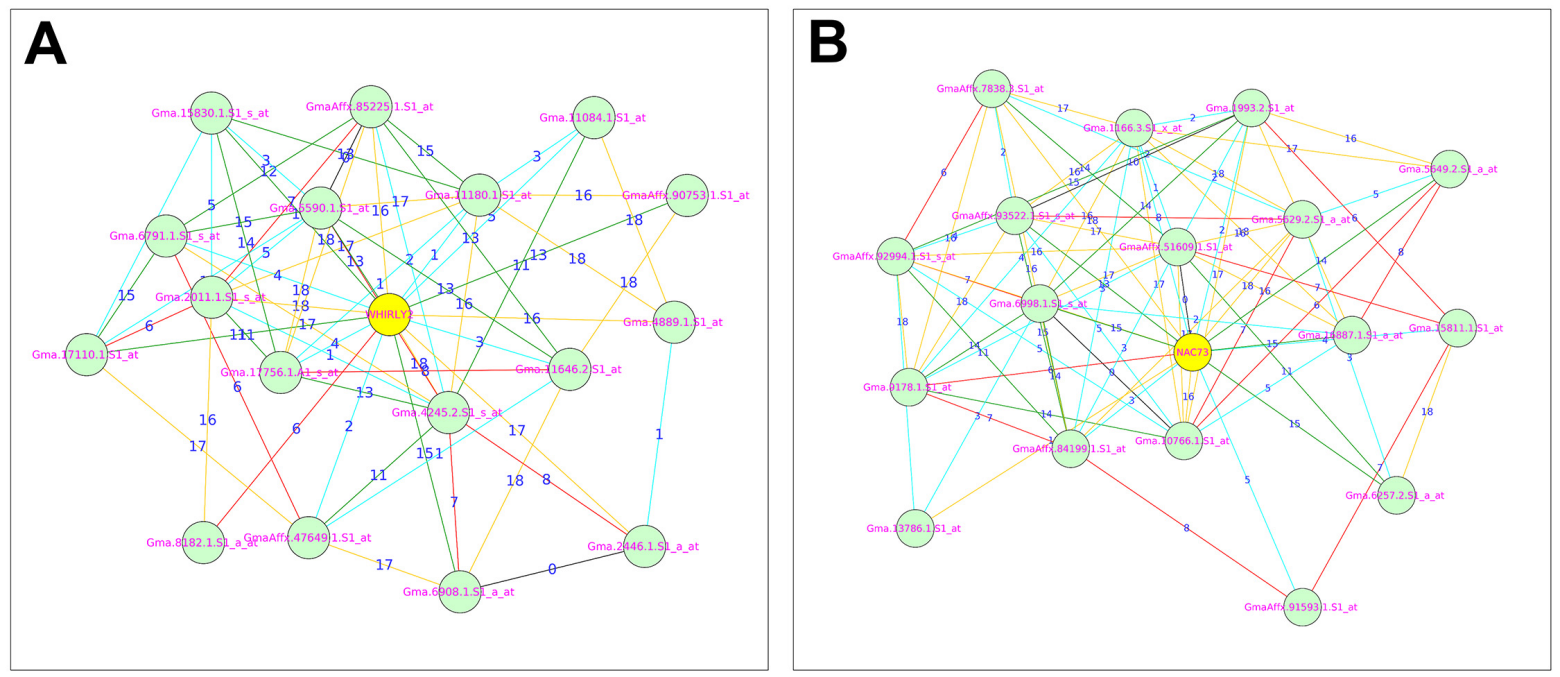
Co-expression relationships of WHIRLY2 and NAC73 genes with their related genes: [A] Shows the co-expression relationships of WHIRLY2 with its related genes. The details of related genes are present in S12 Table. [B] Shows the co-expression relationships of NAC73 with its related genes. The numbers on top of the edges indicates the highest reciprocal rank between node genes. The different edge colors indicates different range of HRR values and as follows: Cyan: HRR1-HRR5; Red: HRR6-HRR10; Green: HRR11-HRR15 and Orange yellow: HRR16-HRR20.

Similarly, module 3 contains two genes encoding two TFs from different families. WHIRLY2 (Glyma03g41270.1) and NAC73 (Glyma15g08480.4). Interestingly, both TFs were up-regulated by the very sub-optimal temperature stress which had the joint highest intramodular connectivity (S13 Table) among identified TFs (Fig 5A and 5B). WHIRLY genes are known to be an important part of disease resistance mechanisms in *Arabidopsis* [54] and induced during salinity stress in *Hordeum vulgare* [52]. So far hundreds of NAC genes have been identified in various plants. Studies on NAC genes revealed that these genes regulate salt and drought tolerance [55, 56]. Interestingly, both WHIRLY2 and NAC73 genes were up-regulated by the very sub-optimal temperature stress which implies that these TFs may have an important role in raising low temperature tolerance by inducing genes associated with cold stimulus response in bambara groundnut. Module 3 is the only module that contains up-regulated TFs indicating that this module may be significantly associated with the very sub-optimal temperature stress. Further investigation is required to confirm whether these two TFs are involved in raising the very sub-optimal temperature stress tolerance in bambara groundnut. Finally, there is only one gene encoding a TF in module 7 (MYB25 Glyma13g09010.1) which is up-regulated by the very sub-optimal temperature stress, consistent with the results of previous studies [43, 52].

## Discussion

Despite being nutritionally rich, bambara groundnut is still cultivated mainly as landraces. Bambara groundnut is well adapted to drought conditions. In contrast, low temperature stress (<T_0_) causes loss of potential yield in bambara groundnut in a similar way to other crops by limiting growth and development [5, 6] and hence restricts farmers’ option for growing the crop, particularly where the temperature is close to T_0_. Hence, the development of low-temperature stress tolerant Bambara groundnut varieties is important in order to provide more options for farmers.

So far most of the existing studies used either array based or sequence based methods to identify stress responsive candidate genes of crops under different kind of stresses [16–19]. But for bambara groundnut so far there is no chip is available and deep sequencing based methods are still expensive and hence we have used a heterologous microarray (Soybean Affymetrix GeneChip^TM^) coupled with network-based analysis to identify the genes and gene modules associated with low temperatures (23^0^ and 18^0^ C) in bambara groundnut. In this study, we developed a computational approach (Fig 1) to identify the genes and gene modules significantly associated with the low temperature stress in bambara groundnut and which can be used to identify the genes and gene modules associated within any particular crop.

In this study, we identified 375 and 659 genes which are differentially expressed in the sub-optimal and the very sub-optimal temperature stresses respectively, 110 of which are commonly shared between stress levels. On average, 41% of annotated genes present in five functional modules of the very sub-optimal temperature stress were significantly enriched with ‘response to stimuli’, ‘response to abiotic stimuli’ and ‘stress response’, genes while none of the modules of the sub-optimal temperature stress treatment were significantly enriched for any stress-related biological process. Among all the annotated genes present in various modules of very sub-optimal temperature stress, there are several important genes which are up-regulated by very sub-optimal temperature stress condition including, HSP, Chaperone DnaJ and WD40 repeat genes. These genes mainly play crucial roles in correct folding of low-temperature stress-accumulated misfolded proteins in order to withstand low-temperatures. Functional annotation of the detected modules identified nine transcription factors (TF) belonging to five transcription factor families in the very sub-optimal temperature stress only (MYB, NAC, WRKY, WHIRLY and GATA). These TFs have already been shown to be significantly associated with various abiotic stresses in various crops [43, 52–56] and therefore, these TFs may regulate downstream target genes involved in various stress responses related to biological processes in bambara groundnut. The identified gene modules and genes could be useful in developing in low-temperature stress tolerant bambara groundnut varieties, via analysis of the existing genetic diversity available within bambara groundnut, through germplasm collections, such as those held at the International Institute for Tropical Agriculture in Nigeria.

## Supporting Information

S1 Fig Validation of 52 DE genes using heatmap. (TIF)

S1 Table List of identified DE genes in Sub_optimal temperature stress. (XLSX)

S2 Table List of identified DE genes in Very sub_optimal temperature stress. (XLSX)

S3 Table List of common DE genes between two stresses. (XLSX)

S4 Table Data used to generate heatmaps. (XLSX)

S5 Table HRR-based co-expression network of Sub_optimal temperature stress. (XLSX)

S6 Table HRR-based co-expression network of Very sub_optimal temperature stress. (XLSX)

S7 Table Detected modules in Sub_optimal temperature stress. (XLSX)

S8 Table Detected modules in Very sub_optimal temperature stress. (XLSX)

S9 Table GO annotation of Sub_optimal temperature stress. (XLSX)

S10 Table Gene-level GO annotation of Sub_optimal temperature stress. (XLSX)

S11 Table GO annotation of Very sub_optimal temperature stress. (XLSX)

S12 Table Gene-level GO annotation of Very sub_optimal temperature stress. (XLSX)

S13 Table List of connected nodes to each of the transcription factor. (XLSX)
