# Supplementary figures and images for: Identification of Gene Modules Associated with Low Temperatures Response in Bambara Groundnut by Network-Based Analysis
Source: PLoS One. 2016 Feb 9;11(2):e0148771. doi: 10.1371/journal.pone.0148771 (PMC4747569; doi:10.1371/journal.pone.0148771)

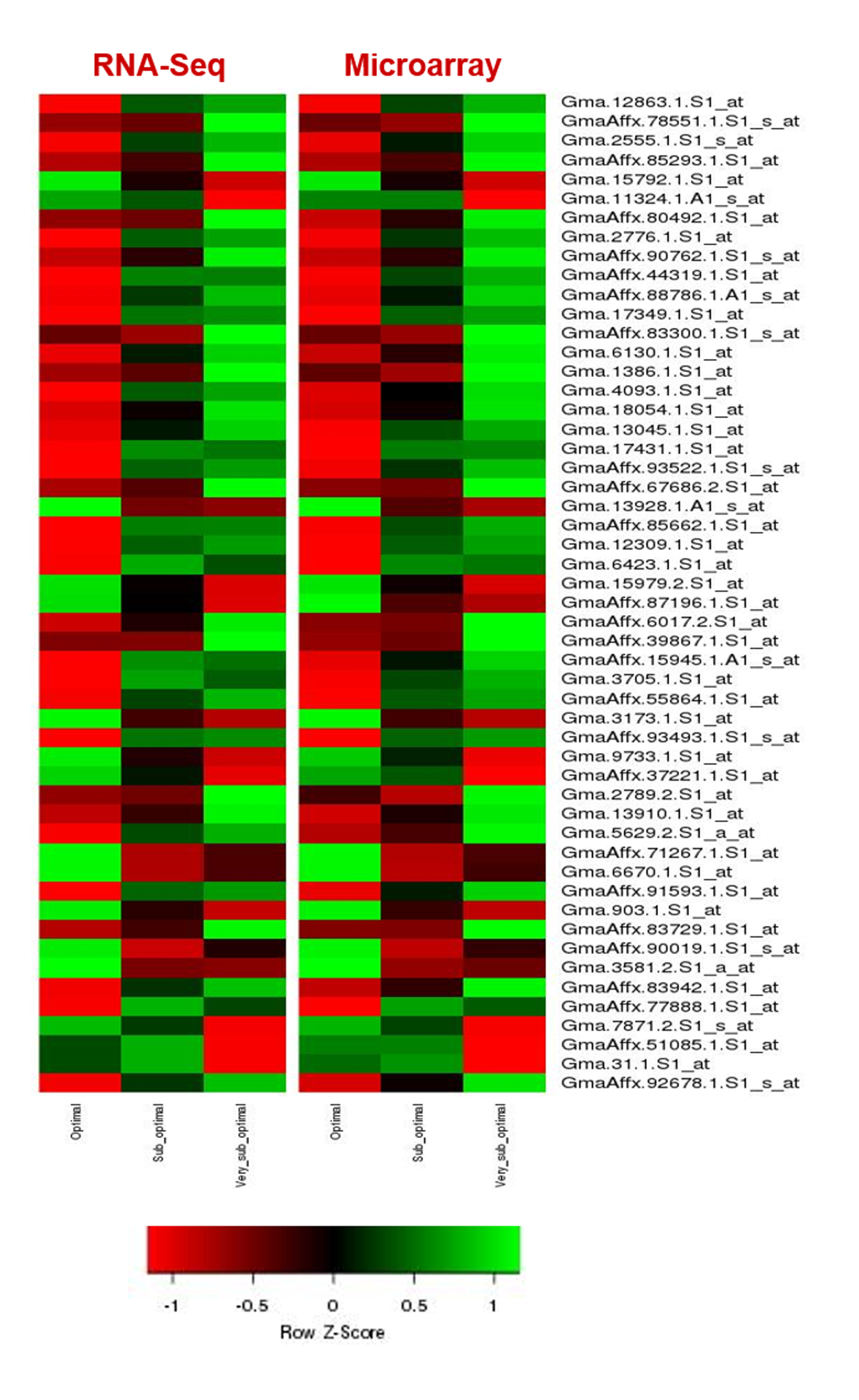

Supplement: S1 Fig — (TIF) [file pone.0148771.s001.tif]
